# Gene model for the ortholog of *Roc1a* in *Drosophila eugracilis*

**DOI:** 10.17912/micropub.biology.001028

**Published:** 2025-03-04

**Authors:** Megan E. Lawson, Isabel G. Wellik, Bridgiet Alvarado, Tanner German, Jeffrey S. Thompson, Lindsey J. Long, Justin R. DiAngelo, Melinda A. Yang, Chinmay P. Rele, Laura K Reed

**Affiliations:** 1 The University of Alabama, Tuscaloosa, AL USA; 2 Denison University, Granville, OH USA; 3 Oklahoma Christian University, Edmond, OK USA; 4 Penn State University, PA USA; 5 University of Richmond, Richmond, VA USA

## Abstract

Gene model for the ortholog of *Regulator of cullins 1a* ( *Roc1a* ) in the *Drosophila eugracilis* Apr. 2013 (BCM-HGSC/Deug_2.0, DeugGB2) Genome Assembly (GenBank Accession: GCA_000236325.2). This ortholog was characterized as part of a developing dataset to study the evolution of the Insulin/insulin-like growth factor signaling pathway (IIS) across the genus *Drosophila* using the Genomics Education Partnership gene annotation protocol for Course-based Undergraduate Research Experiences.

**Figure 1. Roc1a gene model comparison between Drosophila eugracilis and Drosophila melanogaster orthologs:**
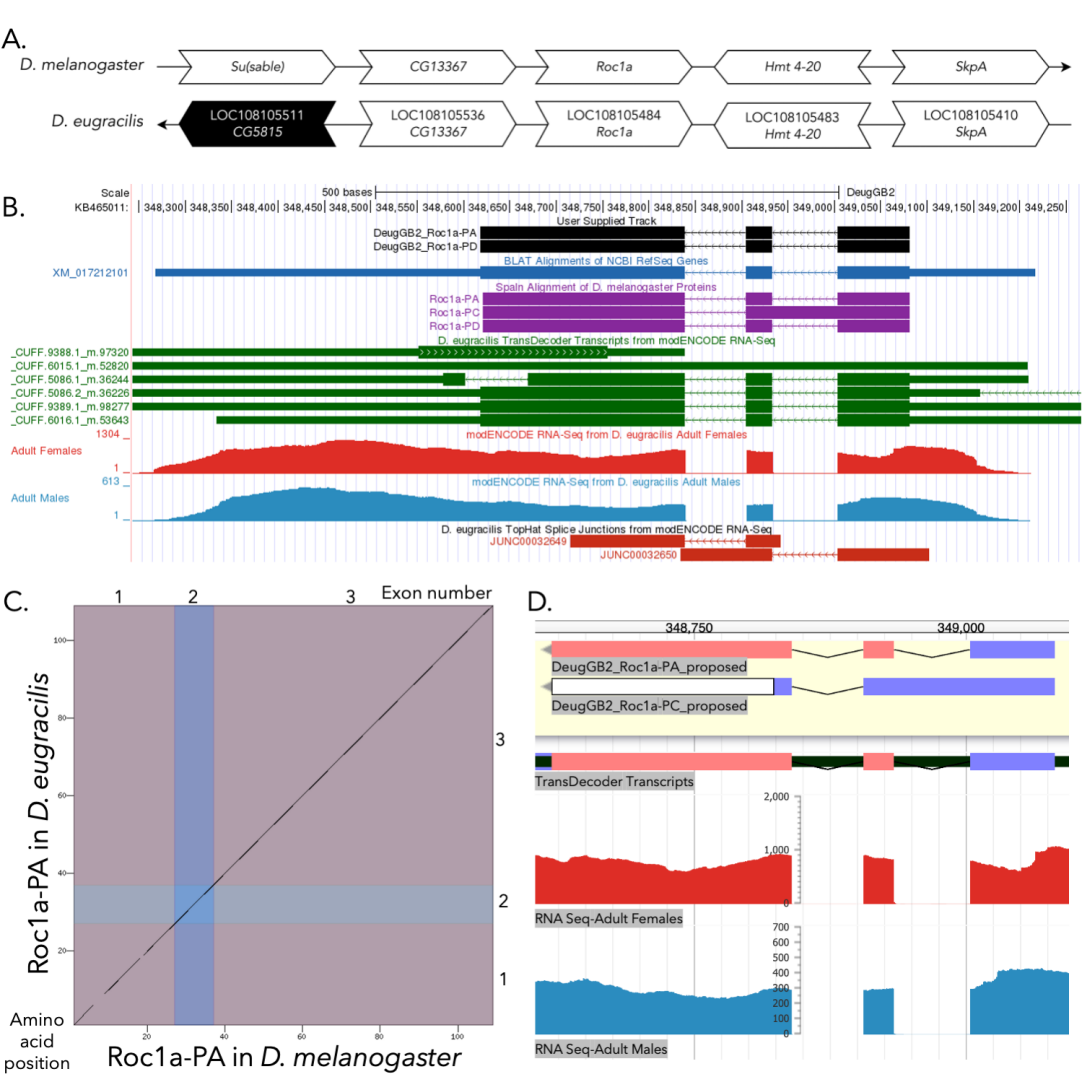
**
(A) Synteny comparison of the genomic neighborhoods for *Roc1a* in *Drosophila melanogaster* and *D.* *eugracilis* .** Thin underlying arrows indicate the DNA strand within which the reference gene– *Roc1a* –is located in *D. melanogaster* (top) and *D. eugracilis* (bottom) genomes. The thin arrow pointing to the right indicates that *Roc1a* is on the positive (+) strand in *D. melanogaster* , and the thin arrow pointing to the left indicates that *Roc1a* is on the negative (-) strand in *D. eugracilis* . The wide gene arrows pointing in the same direction as *Roc1a* are on the same strand relative to the thin underlying arrows, while wide gene arrows pointing in the opposite direction of *Roc1a* are on the opposite strand relative to the thin underlying arrows. White gene arrows in *D. eugracilis* indicate orthology to the corresponding gene in *D. melanogaster* , while black gene arrows indicate non-orthology. Gene symbols given in the *D. eugracilis* gene arrows indicate the orthologous gene in *D. melanogaster* , while the locus identifiers are specific to *D. eugracilis* . **(B) Gene Model in GEP UCSC Track Data Hub** (Raney et al., 2014). The coding-regions of *Roc1a* in *D. eugracilis* are displayed in the User Supplied Track (black); CDSs are depicted by thick rectangles and introns by thin lines with arrows indicating the direction of transcription. Subsequent evidence tracks include BLAT Alignments of NCBI RefSeq Genes (dark blue, alignment of Ref-Seq genes for *D. eugracilis* ), Spaln of *D. melanogaster* Proteins (purple, alignment of Ref-Seq proteins from *D. melanogaster* ), Transcripts and Coding Regions Predicted by TransDecoder (dark green), RNA-Seq from Adult Females and Adult Males (red and light blue, respectively; alignment of Illumina RNA-Seq reads from *D. eugracilis* ), and Splice Junctions Predicted by regtools using *D. eugracilis* RNA-Seq (PRJNA63469). Splice junctions shown have a minimum read-depth of 10 with >1000 supporting reads shown in red. **(C) Dot Plot of Roc1a-PA in *D. melanogaster* ( *x* -axis) vs. the orthologous peptide in *D. eugracilis* ( *y* -axis).** Amino acid number is indicated along the left and bottom; CDS number is indicated along the top and right, and CDSs are also highlighted with alternating colors **. (D) Model of Roc1a-PA compared to Roc1a-PC in Apollo.** A screenshot of the Apollo instance housing the proposed gene model of Roc1a-PA (identical to that of Roc1a-PD) and the likely absent Roc1a-PC model, containing in frame stop codons. The proposed models are shown at the top in the yellow region, while evidence tracks are found below in the white region. CDS reading frames are indicated in blue, green, and red, and implausible CDS sequence warnings (in this case due to the in-frame stop codons) are indicated in white. Evidence tracks from top to bottom include Transcripts and Coding Regions Predicted by TransDecoder and RNA-Seq from Adult Females and Adult Males (red and light blue, respectively; alignment of Illumina RNA-Seq reads from *D. eugracilis* ; PRJNA63469).

## Description

**Table:** 

*This article reports a predicted gene model generated by undergraduate work using a structured gene model annotation protocol defined by the Genomics Education Partnership (GEP; thegep.org) for Course-based Undergraduate Research Experience (CURE). The following information in this box may be repeated in other articles submitted by participants using the same GEP CURE protocol for annotating Drosophila species orthologs of Drosophila melanogaster genes in the insulin signaling pathway.* "In this GEP CURE protocol students use web-based tools to manually annotate genes in non-model *Drosophila* species based on orthology to genes in the well-annotated model organism fruitfly *Drosophila melanogaster* . The GEP uses web-based tools to allow undergraduates to participate in course-based research by generating manual annotations of genes in non-model species (Rele et al., 2023) . Computational-based gene predictions in any organism are often improved by careful manual annotation and curation, allowing for more accurate analyses of gene and genome evolution (Mudge and Harrow 2016; Tello-Ruiz et al., 2019) . These models of orthologous genes across species, such as the one presented here, then provide a reliable basis for further evolutionary genomic analyses when made available to the scientific community.” (Myers et al., 2024) . “ *D. eugracilis* is part of the *melanogaste* r species group within the subgenus *Sophophora* of the genus *Drosophila* (Pélandakis et al., 1993) . It was first described as *Tanygastrella gracilis* by Duda (1924) and revised to *Drosophila eugracilis* by Bock and Wheeler (1972). *D. eugracilis* is found in humid tropical and subtropical forests across southeast Asia (https://www.taxodros.uzh.ch, accessed 1 Feb 2023).” (Morgan et al., 2022) .

We propose a gene model for the *D. eugracilis* ortholog of the *D. melanogaster* *Regulator of cullins 1a* ( *Roc1a* ) gene. The genomic region of the ortholog corresponds to the uncharacterized protein LOC108105484 (RefSeq accession XP_017067590.1 ) in the Apr. 2013 (BCM-HGSC/Deug_2.0) (DeugGB2) Genome Assembly of *D. eugracilis* (GenBank Accession: GCA_000236325.2 ). This model is based on RNA-Seq data from *D. eugracilis* ( PRJNA63469 - Chen et al., 2014) and *Roc1a* in *D. melanogaster* using FlyBase release FB2023_02 ( GCA_000001215.4 ; Larkin et al. 2021; Gramates et al., 2022; Jenkins et al., 2022).

*Roc1a* (also known as *Rbx1, Ring-Box 1, dRbx1, EG:115C2.11 , ROC1* ) is a member of the SCF E3 ubiquitin ligase complex and was originally identified through sequence similarity with vertebrate and yeast homologs and biochemical interaction studies (Bocca et al., 2001) . *Roc1a* deletion mutants are lethal between the first and second larval instars and *Roc1a* mutant clones in imaginal discs have cell proliferation defects (Noureddine et al., 2002) . In addition, Roc1a and other members of the SCF E3 ubiquitin ligase complex function in the pruning of larval neurons by targeting the insulin-responsive kinase Akt for ubiquitination and degradation, thus inhibiting insulin signaling (Wong et al., 2013) .

***Synteny***

The reference gene, *Roc1a* , occurs on chromosome X in *D. melanogaster* and is flanked upstream by *CG13367* and *suppressor of sable* ( *su(sable )* ) and downstream by *Histone methyltransferase 4-20* ( *Hmt4-20* ) and *SKP1-related A* *( SkpA )* . The *tblastn* search of *D. melanogaster* Roc1a-PA (query) against the *D. eugracilis* (GenBank Accession: GCA_000236325.2 ) Genome Assembly (database) placed the putative ortholog of *Roc1a* within scaffold KB465011 (KB465011.1) at locus LOC108105484 ( XP_017067590.1 )— with an E-value of 5e-76 and a percent identity of 96.30%. Furthermore, the putative ortholog is flanked upstream by LOC108105536 ( XP_017067657.1 ) and LOC108105511 ( XP_017067625.1 ), which correspond to *CG13367* and *CG5815* in *D. melanogaster* (E-value: 0.0 and 0.0; identity: 75.82% and 81.68%, respectively, as determined by *blastp* ; Figure 1A, A ltschul et al. 1990). The putative ortholog of *Roc1a* is flanked downstream by LOC108105483 ( XP_017067589.1 ) and LOC108105410 ( XP_017067446.1 ), which correspond to *Hmt4-20* and *SkpA* in *D. melanogaster* (E-value: 0.0 and 4e-117; identity: 84.47% and 98.77%, respectively, as determined by *blastp* ; Figure 1A ). The putative ortholog assignment for *Roc1a* in *D. eugracilis* is supported by the following evidence: the genetic neighborhoods of *Roc1a* in *D. melanogaster* and *D. eugracilis* are completely syntenic with the exception of the second farthest upstream gene, and all *BLAST* search results indicate very high-quality matches.

***Protein Model***

*Roc1a* in *D. eugracilis* has one unique protein-coding isoforms (Roc1a-PA and Roc1a-PD; Figure 1B ) encoded by mRNA isoforms *Roc1a-RA* and *Roc1a-RD* containing three identical protein-coding CDSs. Relative to the ortholog in *D. melanogaster* , the RNA CDS number is conserved for these isoforms, as *Roc1a-RA* and *Roc1a-RD* in *D. melanogaster* also contain three CDSs. However, *D. melanogaster* also has a third isoform with two CDSs, *Roc1a-RC* , that is not present in *D. eugracilis* (see: “special characteristics”). The sequence of Roc1a-PA in *D. eugracilis* has 96.30% identity (E-value: 5e-76) with the protein-coding isoform Roc1a-PA in *D. melanogaster* , as determined by *blastp* ( Figure 1C ) *.* Coordinates of this curated gene model (Roc1a-PA, Roc1a-PD) are stored by NCBI at GenBank/BankIt (accession **BK064556 , BK064557** , respectively **)** . These data are also archived in the CaltechDATA repository (see “Extended Data” section below).

***Special characteristics of the protein model***

mRNA isoform *Roc1a-RC* in *D. melanogaster* is very similar to the other two isoforms, *Roc1a-RA* and *Roc1a-RD* . The difference between these isoforms is that *Roc1a-RA* and *Roc1a-RD* have three CDSs whereas *Roc1a-RC* has two CDSs, with its first CDS (FlyBase ID: 1_2094_0) spanning the length of the first two CDSs of *Roc1a-RA* and *Roc1a-RD* (FlyBase IDs: 2_2094_0 and 3_2094_0) combined (All CDS IDs based on FlyBase release FB2023_02; GCA_000001215.4 ; Larkin et al., 2021). In *D. melanogaster* , the reading frames for the first and second CDSs of *Roc1a-RA* and *Roc1A-RD* are the same, so for isoform *Roc1a-RC* in which the two CDSs are combined, translation is not disrupted. However, in *D. eugracilis,* the reading frames for the first and second CDSs of *Roc1a-RA* and *Roc1A-RD* are different, so the continuous reading frame for the combined CDS in *Roc1a-RC* results in the incorrect reading frame later in translation, and thus the presence of in-frame stop codons in the final CDS of this isoform. This has been highlighted in Figure 1D through the differing CDS colors corresponding to their respective frames, and the white portion of the final CDS in *Roc1a-RC* indicating the presence of in-frame stop codons. This, in combination with the lack of RNA-Seq data and TransDecoder Transcript predictions supporting the existence of *Roc1a-RC* , suggests that *Roc1a-RC* is likely absent from *D. eugracilis* ( Figure 1D ) *.*

## Methods

Detailed methods including algorithms, database versions, and citations for the complete annotation process can be found in Rele et al. (2023). Briefly, students use the GEP instance of the UCSC Genome Browser v.435 ( https://gander.wustl.edu ; Kent WJ et al., 2002; Navarro Gonzalez et al., 2021) to examine the genomic neighborhood of their reference IIS gene in the *D. melanogaster* genome assembly (Aug. 2014; BDGP Release 6 + ISO1 MT/dm6). Students then retrieve the protein sequence for the *D. melanogaster* reference gene for a given isoform and run it using *tblastn* against their target *Drosophila* species genome assembly on the NCBI BLAST server ( https://blast.ncbi.nlm.nih.gov/Blast.cgi ; Altschul et al., 1990) to identify potential orthologs. To validate the potential ortholog, students compare the local genomic neighborhood of their potential ortholog with the genomic neighborhood of their reference gene in *D. melanogaster* . This local synteny analysis includes at minimum the two upstream and downstream genes relative to their putative ortholog. They also explore other sets of genomic evidence using multiple alignment tracks in the Genome Browser, including BLAT alignments of RefSeq Genes, Spaln alignment of *D. melanogaster* proteins, multiple gene prediction tracks (e.g., GeMoMa, Geneid, Augustus), and modENCODE RNA-Seq from the target species. Detailed explanation of how these lines of genomic evidenced are leveraged by students in gene model development are described in Rele et al. (2023). Genomic structure information (e.g., CDSs, intron-exon number and boundaries, number of isoforms) for the *D. melanogaster* reference gene is retrieved through the Gene Record Finder ( https://gander.wustl.edu/~wilson/dmelgenerecord/index.html ; Rele et al *.,* 2023). Approximate splice sites within the target gene are determined using *tblastn* using the CDSs from the *D. melanogaste* r reference gene. Coordinates of CDSs are then refined by examining aligned modENCODE RNA-Seq data, and by applying paradigms of molecular biology such as identifying canonical splice site sequences and ensuring the maintenance of an open reading frame across hypothesized splice sites. Students then confirm the biological validity of their target gene model using the Gene Model Checker ( https://gander.wustl.edu/~wilson/dmelgenerecord/index.html ; Rele et al., 2023), which compares the structure and translated sequence from their hypothesized target gene model against the *D. melanogaster* reference gene model. At least two independent models for a gene are generated by students under mentorship of their faculty course instructors. Those models are then reconciled by a third independent researcher mentored by the project leaders to produce the final model. Note: comparison of 5' and 3' UTR sequence information is not included in this GEP CURE protocol.

## Data Availability

Description: A GFF, FASTA and PEP of the model. Resource Type: Model. DOI: https://doi.org/10.22002/k3acw-7zq30
